# Effects of 25(OH)VD_3_ on Growth Performance, Pork Quality and Calcium Deposit in Growing-Finishing Pigs

**DOI:** 10.3390/ani13010086

**Published:** 2022-12-26

**Authors:** Zeyu Zhang, Gang Zhang, Jindang Cao, Baoqin Qiu, Xiaoyu Qin, Jinbiao Zhao

**Affiliations:** 1 State Key Laboratory of Animal Nutrition, College of Animal Science and Technology, China Agricultural University, Beijing 100193, China; 2 Shandong Haineng Bioengineering Co., Ltd., Rizhao 276800, China

**Keywords:** 25 hydroxyvitamin D_3_, growing-finishing pigs, calcium deposit, antioxidant capacity, growth performance, pork quality

## Abstract

**Simple Summary:**

25-hydroxyvitamin D_3_ [25(OH)VD_3_] plays an important role in regulating calcium and phosphorus metabolism via upregulating the expression of calcium ion channel proteins, resulting in improved bone quality. Many publications have reported that dietary supplementation of 25(OH)VD_3_ could improve the antioxidase activity and immune function of weaned pigs. However, evidence has proven that the biological response of 25(OH)VD_3_ on pig nutrition depends on the inclusion doses in the diets. This study was conducted to explore the roles of 25(OH)VD_3_ with different inclusion doses in growing-finishing pigs.

**Abstract:**

The study was conducted to evaluate the effects of 25(OH)VD_3_ with different inclusion levels of 0, 25, 50 and 75 μg/kg in the diet on growth performance, nutrient digestibility, bone properties and pork quality in growing-finishing pigs. The results showed that the average daily gain (*p* < 0.05) and body weight (*p* < 0.10) of pigs showed a trend of increasing quadratically as inclusion levels of 25(OH)VD_3_ increased. Dietary supplementation of 50 μg/kg 25(OH)VD_3_ increased calcium digestibility compared with the 0 μg/kg group (*p* < 0.05), and calcium and phosphorus digestibility increased quadratically as inclusion levels of 25(OH)VD_3_ increased (*p* < 0.05). Dietary supplementation of 50 μg/kg 25(OH)VD_3_ increased concentrations of polyunsaturated fatty acids, and decreased contents of saturated and monounsaturated fatty acids in the longissimus dorsi of pigs (*p* < 0.05). The addition of 25, 50 and 75 μg/kg 25(OH)VD_3_ to the diet increased breaking strength and bone stiffness in the tibia compared with the 0 μg/kg group (*p* < 0.05). Dietary supplementation of 50 μg/kg 25(OH)VD_3_ improved the activities of superoxide dismutase (SOD) and catalase (CAT), and increased the messenger RNA (mRNA) expression of Cu/Zn SOD in the longissimus dorsi compared with the 0 μg/kg group (*p* < 0.05). Supplementing 50 μg/kg 25(OH)VD_3_ improved the mRNA expression of calcium-binding protein D9k (CaBP-D9k) and D28k (CaBP-D28K) in the liver compared with the 0 μg/kg 25(OH)D_3_ group (*p* < 0.05). In conclusion, a diet with an added dose of 50 μg/kg 25(OH)VD_3_ showed a greatest growth performance of growing-finishing pigs, and 25(OH)VD_3_ enhanced calcium deposition and antioxidant capacity in the longissimus dorsi, which may be associated with improved expression of calcium ion channel proteins.

## 1. Introduction

Vitamin D is one of the most important nutrients for maintaining the normal growth performance of pigs. A deficiency or metabolic abnormalities of vitamin D are known to be associated with myopathies such as muscle weakness, hypotonia, and skeletal muscle atrophy [1]. In practice, the common forms of vitamin D supplied in pig feed are vitamin D_3_ (VD_3_) and 25-hydroxyvitamin D_3_ [25(OH)VD_3_]. Vitamin D_3_ is a fat-soluble vitamin with a cholecalciferol-like biological activity, and 25(OH)D_3_ can be synthesized by VD_3_ in the liver [2]. However, the primary active form of vitamin D is 1,25-hydroxyvitamin D_3_ [1,25(OH)VD_3_], which is converted from VD_3_ and 25(OH)VD_3_ in the liver and kidney [3]. 25-hydroxyvitamin D_3_ has stronger physiological activity and can promote animal bone development, improve the feed conversion ratio and enhance disorder resistance [4]. Dietary supplementation of 25(OH)VD_3_ can not only shorten the metabolic process of VD_3_ in the body, but also avoid the effects of intestinal injury and liver and kidney dysfunction in pigs [5]. In addition, 25(OH)VD_3_ plays an important role in regulating calcium and phosphorus metabolism via upregulating the expression of calcium ion channel proteins, resulting in improved bone quality [6,7]. Many publications have reported that dietary supplementation of 25(OH)VD_3_ could improve the antioxidase activity and immune function of weaned pigs [8]. However, evidence has proven that the biological response of 25(OH)VD_3_ on pig nutrition depends on the inclusion doses in the diets [9]. A suitable inclusion level of 25(OH)VD_3_ could elicit osteoblast proliferation and improve bone formation and mineralization via activating the signaling pathway of vitamin D receptors [10], but excessive supplementation of 25(OH)VD_3_ caused bone catabolism and inhibited bone mineralization [11]. Therefore, the relationship between different inclusion levels of 25(OH)VD_3_ and pig nutrition should be further explored.

In our previous publication, the effects of different inclusion levels of 25(OH)VD_3_ on weanling pigs were explored, and the results indicated that a dietary addition of 50 μg/kg 25(OH)VD_3_ supplementation partly improved the growth performance, immune function, antioxidant status, intestinal morphology and bone properties of weaned piglets compared with dietary treatments of 25 μg/kg and 75 μg/kg 25(OH)VD_3_ [8]. However, the roles of 25(OH)VD_3_ with different dietary inclusion doses of 25(OH)VD_3_ on the nutrition of growing-finishing pigs have been unclear. Therefore, we hypothesized that the beneficial effects of 25(OH)VD_3_ on the growth performance and antioxidase activity of growing-finishing pigs, and the responses of 25(OH)VD_3_ on the pigs, are associated with the inclusion doses of 25(OH)VD_3_. In the present study, the effects of different inclusion levels of 25(OH)VD_3_ on growth performance, pork quality, antioxidant capacity, and nutrient digestibility in growing-finishing pigs were explored. 

## 2. Materials and Methods 

### 2.1. Animals and Diets

A total of 192 growing-finishing pigs (Duroc × Landrace × Yorkshire) with an initial body weight of 46.5 ± 2.7 kg were randomly allocated into 4 dietary treatments with 6 replicates per treatment, and each replicate included 8 pigs (4 boars and 4 gilts). The diets were formulated to provide adequate nutrition in three phases, mixed, and then supplemented with 0, 25, 50 and 75 μg/kg 25(OH)VD_3_ (Table 1). For each diet within each phase, the 25(OH)VD_3_ was first mixed with dicalcium phosphate and limestone, and then added to wheat bran to help ensure the distribution before the diets were completed with the corn and soybean meal. Subsamples of the diets were collected using multipoint sampling. All diets were supplemented with 25 μg/kg vitamin D_3_ from the premix, and the analyzed concentrations of vitamin D were 30.5, 55.1, 80.9 and 105.3 μg/kg in the diets supplemented with 0, 25, 50 and 75 μg/kg 25(OH)VD_3_, respectively. The feeding trial lasted 84 days and was conducted at the Swine Research Unit of China Agricultural University (Hebei, China). The addition of 0.3% chromic oxide as an indigestible marker was used to determine nutrient digestibility in the last 14 days of the feeding trial. The ingredient composition and nutritive levels of the diets with an inclusion level of 0 μg/kg 25(OH)D_3_ in the different phases of the pigs are shown in Table 1. Vitamin and mineral premixes were supplemented to meet the nutrient requirements of growing-finishing pigs according to the NRC (2012) [12]. During the whole feeding trial, all the pigs were provided *ad libitum* access to water and rations. The piglets in each replicate were housed in pens (1.5 × 1.2 × 1.0 m^3^), and the humidity and temperature of the feeding room were controlled automatically at 45–55% and 18 °C–22 °C. All pigs were dewormed according to the routine procedures of the pig farm. At days 28, 56 and 84, the piglets were weighed and the feed consumption was recorded to calculate the average daily gain (ADG) and average daily feed intake (ADFI). A ratio of ADG to ADFI (G: F) for the pigs was calculated.

### 2.2. Sample Collection

At the end of the experiment, 300 g of fresh feces were collected from each replicate (pen) and then dried in the oven at 65 °C for 72 h. Considering the best growth performance was observed in the 50 μg/kg 25(OH)VD_3_ group compared with the other dietary treatments with different inclusion doses of 25(OH)VD_3_ at the end of the feeding trial, one barrow close to the average body weight of each replicate in the 0 μg/kg and 50 μg/kg 25(OH)VD_3_ groups was slaughtered to collect samples of longissimus dorsi, liver and tibia using an electric shock slaughter method. The carcass traits were measured immediately after slaughter, and the longissimus dorsi at the left 10th to 11th ribs were taken for determining pH, meat color and drip loss at 45 min and 24 h after the slaughter. In addition, samples of longissimus dorsi were frozen at −20 °C to analyze compositions of amino acids and fatty acids. Samples of the liver were taken and stored at −80 °C after quick-freezing in liquid nitrogen for determining the expression of antioxidase activity and calcium iron channel proteins. The tibia was stored at 4 °C for the further determination of skeletal properties after removing the fat and muscle.

### 2.3. Nutrient Digestibility

Apparent total tract digestibility (ATTD) of the nutrients were calculated using the indicator method as follows:

ATTD (%) = 100 − (RC × FN)/(FC × RN) × 100

where RC is the concentration of chromium in the ration (%), RN is the concentration of nutrients in the ration (%), FN is the concentration of nutrients in the feces (%), FC is the concentration of chromium in the feces (%), and DN is the concentration of nutrients in the diet (%).

### 2.4. Chemical Analysis

The diets and dried fecal samples were crushed and passed through a 60-mesh sieve for analysis. Dry matter (DM; Method 934.01), ether extract (EE; Method 920.39), crude protein (CP; Method 990.03), and chromium (Method 990.08) in the diets and feces were analyzed in duplicate [13].

### 2.5. Inflammatory Cytokines and Antioxidant Capacity in the Liver

The levels of antioxidant parameters including superoxide dismutase (SOD), glutathione peroxidase (GSH-Px), and catalase (CAT) were determined using assay kits following the manufacturer’s instructions (Nanjing Jiancheng Bioengineering Institute, Nanjing, China). Concentrations of interleukin-1β (IL-1β), interleukin-6 (IL-6), interleukin-10 (IL-10), interleukin-12 (IL-12), and tumor necrosis factor (TNF-α) were determined using commercially available porcine ELISA kits (Nanjing Jiancheng Bioengineering Institute, Nanjing, China).

### 2.6. Carcass Traits

After slaughter, the carcass of pigs was divided according to the conventional procedure, and the carcass weight was measured after removing the head, hooves, tail and internal organs while the kidneys and leaf fat were retained. The oblique length of the carcass was the length from the inner edge of the union of the first rib and sternum to the anterior edge of the pubic symphysis. The backfat thickness at the 10th rib of the left half of the carcass was measured with vernier calipers, while the length and width of the longissimus dorsi were determined. The slaughter rate and eye muscle area were calculated as follows:

Slaughter rate (%) = the carcass weight/the live weight before slaughter × 100

Eye muscle area (cm^2^) = length × width × 0.7

Meat quality including shear force, cooking loss, drip loss, pH_45min_, pH_24h_, lightness (L*), redness (a*) and yellowness (b*) were determined immediately after the slaughter. The pH of the longissimus dorsi was determined 45 min and 24 h after the slaughter using a portable acidity meter (OPTP-STAR, Meister, Germany). The drip loss was determined according to the method described in a previous study [14]. Briefly, samples of approximately 30 g were cut from the longissimus dorsi, and then were stored in covered plastic boxes over sieved plastic racks for 48 h at 2 to 4 °C. After storage, the samples were re-weighed and the difference in weight was used to determine drip loss. In addition, about 100 g of samples from the longissimus dorsi were packaged under vacuum and cooked in a water bath at 80 °C for 45 min. The difference in weight before and after cooking was used to determine cooking loss. Subsequently, the samples after determination of the cooking loss were blotted dry and cooled for 10 h. A hollow metal probe was used to cut 10 subsamples from each block of meat along the length of the fiber direction, and the subsamples were used to determine the shear force by using an Instron machine (Stable Micro Systems Ltd., Surrey, UK).

### 2.7. Contents of Amino Acids (AA) and Fatty Acids (FA) in the Longissimus Dorsi

The longissimus dorsi was cut into 2 mm of thin slices to weight by using an aluminum box, and then freeze-dried (Tofflon Freezing Drying Systems, Shanghai, China). Lyophilized meat was subsequently crushed into powder to analyze the intramuscular fat concentration by using the Soxhlet petroleum ether extraction (XT15 Extractor, Ankom Technology Corp., Macedon, NY, USA) as described by Zhang et al. [15]. Concentrations of fatty acids were determined using classical gas chromatography (6890 Series, Agilent Technologies, Wilmington, DE, USA) as described in a previous report [16]. Moreover, methionine and cysteine were determined as methionine sulphone and cysteic acid using an amino acid analyzer (Hitachi L-8900, Tokyo, Japan) after cold performic acid oxidation overnight and hydrolyzing with 7.5 mol/L HCl at 110 °C for 24 h. Tryptophan was determined using high performance liquid chromatography (Agilent 1200 Series, Santa Clara, CA, USA) after LiOH hydrolysis for 22 h at 110 °C.

### 2.8. Skeletal Characteristics

Bone density was determined according to Archimedes’ principle as follows:

Bone density = [A/(A − B)] × *p*

where A is the weight after leaving the water surface, B is the weight when fully immersed in distilled water, and *p* is the density of the distilled water.

The bone mechanical properties were determined using a three-point bending test with an MTS-810 universal tensile tester (MTS Systems Corporation, Eden Prairie, MN, USA) [17]. The details on the analysis procedures of the bone mechanical properties followed those of a previous study [18].

### 2.9. Quantification PCR Analysis

Total ribonucleic acid (RNA) was extracted from the liver by the RN01-TRIpure Reagent (Aidlab Biotechnologies Co., Ltd., Beijing, China) according to the manufacturer’s protocol. The extracted RNA was quantified using NanoDrop 2000 (Thermo Fisher Scientific, MA, USA), and then diluted to the same concentration in each sample. The complementary deoxyribonucleic acid (cDNA) was produced using a reverse transcription kit (Takara, Kusatsu, Shiga, Japan). Quantitative polymerase chain reaction (PCR) was performed on a Riche light cycler 96 Real-Time PCR System (Roche, CA, USA). The PCR reaction procedure was: 5 min pre-denaturation at 95 °C, 20 s at 95 °C, 30 s at 60 °C, 40 cycles, and a melting curve of 65–90 °C. The primer sequences of each gene are shown in Supplementary Table S1. The relative expression of target genes to that of a housekeeping gene (GAPDH) was calculated using the 2^−ΔΔCt^ method.

### 2.10. Statistical Analysis

Normality was verified and outliers were identified using the UNIVARIATE procedure of SAS 9.4 (SAS Institute, Cary, NC, USA), and a general linear model (GLM) of SAS was used to analyze the observations with the pen (replicate) as the experimental unit. An observation was considered an outlier if the value was more than three standard deviations away from the grand mean, but no outliers were observed in the study. The different dietary treatments were considered fixed effects, and the experimental animal status and feeding management (such as weaning age, humidity and temperature of the pens) were random effects. Means were separated using the LSMEANS statement and adjusted using Tukey’s multiple comparison tests. Polynomial contrast was conducted to determine the linear and quadratic effects of inclusion doses. A significant difference was considered when *p* < 0.05 and a tendency when 0.05 < *p* < 0.10.

## 3. Results

### 3.1. Growth Performance

There were no significant differences in the body weight (BW), ADG, ADFI and G: F of the pigs among different dietary groups (Table 2). However, the ADG in days 28–56 and days 1–84 showed a quadratic response as dietary 25(OH)D_3_ levels increased from 0 to 75 μg/kg (*p* < 0.05) with a maximum at 50 ug/kg. There was a trend that pig BW on day 56 and day 84 was maximized at 50 ug/kg and responded quadratically as inclusion levels of 25(OH)D_3_ increased (*p* < 0.10).

### 3.2. Nutrient Digestibility

There were no significant differences in the digestibility of DM, EE, CP, calcium and phosphorus in pigs among different dietary groups (Table 3). However, the digestibility of calcium and phosphorus showed a quadratic response with a numerical maximum at 50 μg/kg to dietary 25(OH)D_3_ levels from 0 to 75 μg/kg (*p* < 0.05).

### 3.3. Carcass Traits and Pork Quality

There were no significant differences in carcass traits (Table 4) and the composition of amino acids (Table 5) in the longissimus dorsi of the pigs between the 0 μg/kg and 50 μg/kg 25(OH)D_3_ groups. The dietary inclusion of 50 μg/kg 25(OH)VD_3_ decreased the concentrations of saturated and monounsaturated fatty acids in the longissimus dorsi of pigs, but increased the proportion of polyunsaturated fatty acids (*p* < 0.05; Table 6). In addition, providing 50 μg/kg 25(OH)VD_3_ increased a ratio of ∑ *n*-6 polyunsaturated fatty acid (PUFA) to ∑ *n*-3 PUFA and decreased the ratio of polyunsaturated fatty acids to saturated fatty acids (*p* < 0.05).

### 3.4. Skeletal Characteristics

There were no significant differences in phosphorus and bone mineral contents, bone mineral density and destruction deflection between the 0 μg/kg and 50 μg/kg 25(OH)D_3_ groups (Table 7). There was a trend that 50 μg/kg 25(OH)VD_3_ increased calcium concentration of the tibia (*p* < 0.10). The group of 50 μg/kg 25(OH)D_3_ significantly increased the break strength and stiffness in the tibia of pigs compared with the 0 μg/kg 25(OH)D_3_ group (*p* < 0.05).

### 3.5. Inflammatory Cytokines

**Figure 1:**
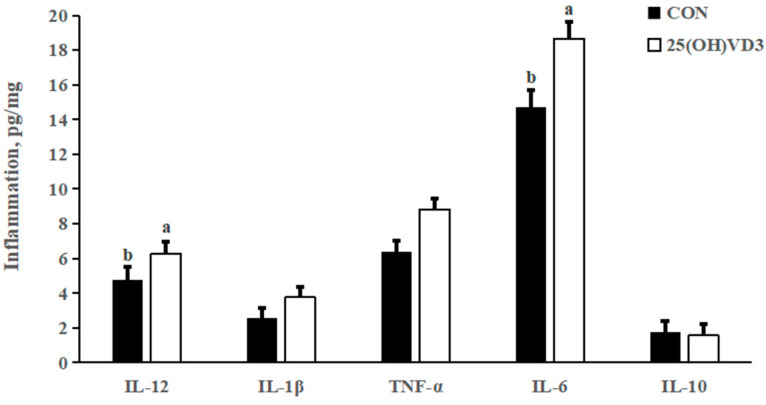
Effects of 50 µg/kg 25(OH)VD_3_ on inflammatory cytokines concentration in the liver of growing-finishing pigs. Note: Superscript letters within the bar chart indicate significant differences (*p* < 0.05), *n* = 6. 25(OH)VD_3_, 25-hydroxyvitamin D_3_.

There were no significant differences in the concentrations of IL- 1β, TNF-α and IL-10 in the liver between the 0 μg/kg and 50 μg/kg 25(OH)D_3_ groups (Figure 1). However, provision of 50 μg/kg 25(OH)VD_3_ increased the concentrations of IL-12 and IL-6 in the liver of pigs compared with the 0 μg/kg 25(OH)VD_3_ group (*p* < 0.05).

### 3.6. Antioxidase Activity

**Figure 2:**
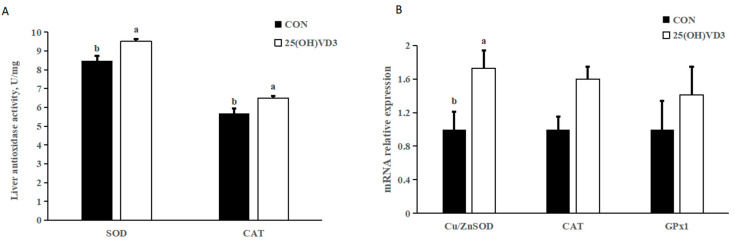
Effects of 50 µg/kg 25(OH)VD_3_ on antioxidase activity in the liver of growing-finishing pigs. (**A**) Concentration of liver antioxidase activity. (**B**) mRNA expression of antioxidase genes. Note: Superscript letters within the bar chart indicate significant differences (*p* < 0.05), *n* = 6. 25(OH)VD_3_, 25-hydroxyvitamin D_3_.

Providing 50 μg/kg 25(OH)VD_3_ increased the concentrations of SOD and CAT and the mRNA expression of Cu/Zn SOD in the liver of pigs compared with the 0 μg/kg 25(OH)D_3_ group (*p* < 0.05; Figure 2). There were no differences in the activity of the mRNA expression of CAT and GPx1 between the 0 μg/kg and 50 μg/kg 25(OH)D3 groups.

### 3.7. mRNA Expression of Calcium Ion Channel Proteins

**Figure 3:**
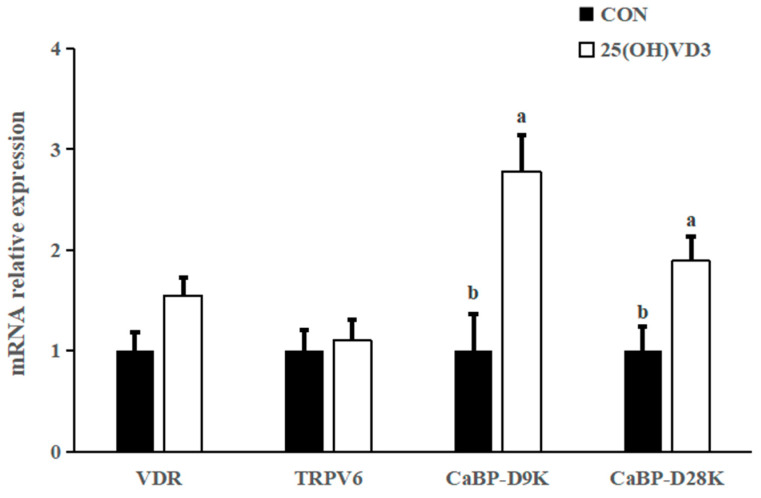
Effects of 50 µg/kg 25(OH)VD3 on the mRNA expression of calcium ion channel proteins in the liver of finishing pigs. Note: Superscript letters within the bar chart indicate significant differences (*p* < 0.05), *n* = 6. 25(OH)VD_3_, 25-hydroxyvitamin D_3_.

The dietary addition of 50 μg/kg 25(OH)VD_3_ tended to increase the mRNA expression of vitamin D receptor (VDR) (*p* < 0.10), calcium-binding protein D9k (CaBP-D9k) and calcium-binding protein D28k (CaBP-D28K) in the liver of pigs compared with the 0 μg/kg 25(OH)VD_3_ group (*p* < 0.05; Figure 3). There was no difference in the mRNA expression of TRPV6 between the 0 μg/kg and 50 μg/kg 25(OH)VD_3_ groups.

## 4. Discussion

### 4.1. Effects of Different Inclusion Levels of 25(OH)VD_3_ on Growth Performance and Nutrient Digestibility

In the present study, the ADG of pigs in days 28–56 and days 1–84 and pig body weight on day 56 and day 84 showed a quadratic change as dietary 25(OH)VD_3_ levels increased from 0 to 75 μg/kg. The pigs had the greatest growth performance when the inclusion level of 25(OH)VD_3_ was 50 μg/kg in addition to the 25 ug/kg already in the diet. The above results were consistent with those in a previous study that showed a quadratic response of dietary 25(OH)VD_3_ levels on the ADG and body weight of weanling piglets, and an inclusion level of 50 μg/kg 25(OH)VD_3_ was recommended [8]. The results of a greatest growth performance in the 50 μg/kg 25(OH)VD_3_ for weanling piglets and growing-finishing pigs are consistent, indicating that responses of 25(OH)VD_3_ at an inclusion level of 50 μg/kg 25(OH)VD_3_ on pigs’ performance has no correlation to the growth phase of pigs from 8 kg to 120 kg, but further study should be conducted to confirm the above conclusion. In addition, the dietary supplementation of 25(OH)VD_3_ with different inclusion doses affected the quadratic digestibility of calcium and phosphorus in growing-finishing pigs, and the pigs in the group of 50 μg/kg 25(OH)VD_3_ showed the greatest digestibility of calcium and phosphorus in our study. The above result agreed with those in many previous studies, in which the dietary supplementation of 25(OH)VD_3_ increased calcium and phosphorus digestibility in sows and growing pigs [19]. Many previous studies have reported that providing an additional 50 μg/kg 25(OH)VD_3_ increased the digestibility of calcium and phosphorus, but did not affect the growth performance of growing-finishing pigs [20,21]. The reasons for the increased digestibility of calcium and phosphorus are associated with the improved activity of phytase and the upregulated expression of calcium ion channel proteins [21,22]. In addition, a previous study reported that dietary total phosphorus levels have marginal effects on the ileal digestibility of phosphorus in growing pigs [23]. Therefore, it is a useful strategy for supplementing the 25(OH)VD_3_ to increase the utilization of dietary phosphorus.

### 4.2. Effects of 50 μG/KG 25(OH)VD_3_ on Carcass Traits and Pork Quality

There were no significant differences in the carcass traits in the longissimus dorsi of pigs between the 0 μg/kg and 50 μg/kg 25(OH)VD_3_ groups, although the dietary supplementation of 50 μg/kg 25(OH)D_3_ increased the antioxidase activity in the liver, which was consistent with a previous report [24]. Those results indicated that 25(OH)VD_3_ has no influences on carcass traits, although 25(OH)VD_3_ can improve growth performance. A previous study reported that an increased activity of antioxidases improved the pork quality of finishing pigs [25]. In addition, samples from the pigs in the group of 50 μg/kg 25(OH)VD_3_ had decreased concentrations of saturated and monounsaturated fatty acids in the longissimus dorsi of the pigs, but an increased proportion of polyunsaturated fatty acids in the present study. However, a previous study reported that 50 μg/kg 25(OH)VD_3_ to the sows had no effect on the concentration of fatty acids in the sow’s milk and in the tibial and femoral cortical bones of new-born piglets [26]. The dietary addition of 50 μg/kg 25(OH)VD_3_ increased a ratio of ∑*n*-6 PUFA to ∑*n*-3 PUFA and decreased a ratio of polyunsaturated fatty acids to saturated fatty acids, which indicated that 25(OH)VD_3_ promoted the synthesis of polyunsaturated fatty acids in the growing-finishing pigs. The increased synthesis of polyunsaturated fatty acids induced by 25(OH)VD_3_ may be associated with the upregulating expression of calcium ion channel proteins, but the potential mechanism should be further clarified.

### 4.3. Effects of 50 μG/KG 25(OH)VD_3_ on Immune Function

In our previous study, a dietary supplementation of 50 μg/kg 25(OH)VD_3_ increased the concentrations of IL-1β in the jejunum and colon of weanling piglets. In addition, Zhang et al. [27] reported that 50 μg/kg 25(OH)VD_3_ added in the diet increased serum concentrations of IgM and IgG in weanling pigs. The above results indicate that 25(OH)VD_3_ improves the immune responses of pigs in resisting infection by pathogens, which is consistent with the results of the present study that dietary supplementation of 50 μg/kg 25(OH)VD_3_ increased concentrations of IL-12 and IL-6 in the liver of growing-finishing pigs. However, 25(OH)VD_3_ could decrease the concentrations of TNF-α, IL-2 and IL-6 through suppressing the activation of T cells [28]. Varying responses of 25(OH)VD_3_ on the concentrations of inflammatory cytokines should be associated with the healthy status of pigs.

### 4.4. Effects of 50 μG/KG 25(OH)VD_3_ on Antioxidant Capacity

The pigs that were fed an additional 50 μg/kg 25(OH)VD_3_ had increased concentrations of SOD and CAT and mRNA expression of Cu/Zn SOD in the liver compared with the pigs from the 0 μg/kg 25(OH)VD_3_ group, which indicates that 25(OH)VD_3_ can improve antioxidant capacity in growing-finishing pigs. The present results agree with those from many previous studies in which the dietary supplementation of 50 μg/kg 25(OH)VD_3_ increased the activity of T-AOC and GSH-Px in the serum of weanling piglets. The improved antioxidant capacity may result in an increase in the a* value of the carcass trait by reducing the oxidation of pork after the slaughter [29]. However, although 50 μg/kg 25(OH)VD_3_ increased the antioxidant capacity of the pigs, no significant improvements on the carcass trait were observed in our study.

### 4.5. Effects of 50 μG/KG 25(OH)VD_3_ on Bone Characteristics and Expression of Calcium Ion Channel Protein

Vitamin D_3_ can induce osteoblast proliferation through the VDR signaling pathway and promote bone formation and mineralization [30]. However, high-dose vitamin D_3_ supplementation will promote bone decomposition and absorption and inhibit bone mineralization [31]. In our study, there was a trend that 50 μg/kg 25(OH)VD_3_ increased the calcium concentration of the tibia. Furthermore, the addition of 50 μg/kg 25(OH)VD_3_ increased the bone breaking strength and stiffness in the tibia of pigs compared with the 0 μg/kg 25(OH)VD_3_ group. A previous study showed that dietary supplementation of 50 μg/kg 25(OH)VD_3_ fed to sows from late gestation to weaning increased calcium content, bone density and breaking strength in the tibias and femurs of sows [5]. Dietary supplementation of 25 μg/kg 25(OH)VD_3_ increased bone mineral content and breaking strength in the femur of weanling piglets in a previous study [8].

In addition, 50 μg/kg 25(OH)VD_3_ increased the mRNA expression of VDR, CaBP-D9K and CaBP-D28K in the tibia of pigs compared with the 0 μg/kg 25(OH)VD_3_ group. The above publication reported 50 μg/kg 25(OH)VD3 upregulated mRNA expression of duodenal VDR, transient receptor potential vanilloid 6 (TRPV6) and CaBP-D9k in sows, as well as ileal VDR and claudin-2, colonic VDR and CaBP-D9k in new-born piglets. Overall, a diet supplementing 50 μg/kg 25(OH)VD3 can improve bone characteristics and calcium deposition through upregulating the mRNA expression of calcium ion channel proteins.

## 5. Conclusions

The growth performance of growing-finishing pigs showed a quadratic change as inclusion levels of 25(OH)VD_3_ increased from 0 to 75 μg/kg, and the diet with an inclusion level 50 μg/kg 25(OH)VD_3_ showed a greater growth performance than 0, 25 and 75 μg/kg. A diet supplementing 50 μg/kg 25(OH)VD_3_ increased the concentrations of polyunsaturated fatty acids, but did not affect the amino acid concentrations in the longissimus dorsi of pigs. In addition, a diet supplementing 50 μg/kg 25(OH)D_3_ improved bone characteristics and calcium deposition through upregulating the mRNA expression of calcium ion channel proteins, resulting in an increased digestibility of calcium, as well as an improvement in liver antioxidant capacity.

## Figures and Tables

**Table 1:** Dietary formulation and analyzed or calculated nutrient levels (%) for each phase.

	Growth Phases of Pigs		
Items	50–75 kg	75–100 kg	100 kg–Harvest
Ingredients			
Corn	69.27	74.91	80.05
Soybean meal	20.00	15.00	10.50
Wheat bran	8.00	7.50	7.00
Dicalcium phosphate	0.18	0.08	-
Limestone	1.20	1.18	1.15
Salt	0.50	0.50	0.50
L-Lysine HCl, 78.8%	0.25	0.24	0.22
DL-Methionine	0.03	0.02	0.01
L-Threonine	0.05	0.05	0.05
L-Tryptophan	0.02	0.02	0.02
Premix ^1^	0.50	0.50	0.50
Analyzed nutrient levels			
Crude protein	15.50	13.70	12.10
Calcium	0.82	0.81	0.82
Total phosphorus	0.63	0.59	0.61
Calculated nutrient levels, %			
Digestible energy, Mcal/kg	3.40	3.40	3.40
Available phosphorus	0.19	0.17	0.15
Standardized ileal digestible lysine	0.86	0.73	0.62
Standardized ileal digestible methionine	0.24	0.22	0.19
Standardized ileal digestible threonine	0.53	0.47	0.41
Standardized ileal digestible tryptophan	0.16	0.14	0.11

Note: ^1^ Premix provided per kg diet: vitamin A, 5600 IU; vitamin D_3_, 1000 IU; vitamin E, 21.6 IU; vitamin K_3_, 1.8 mg; vitamin B_6_, 1.8 mg; vitamin B_12_, 12 μg; riboflavin, 4.0 mg; thiamine, 0.88 mg; niacin, 20 mg; pantothenic acid, 10 mg; folic acid, 0.4 mg; biotin, 40 μg; choline chloride, 320 mg; Mg, 10 mg (MgO); Fe, 100 mg (FeSO_4_·H_2_O); Zn, 125 mg (ZnO); Cu, 15 mg (CuSO_4_·5H_2_O); I, 50 mg (CaI_2_); Se, 0.30 mg (Na_2_SeO_3_).

**Table 2:** Effects of 25(OH)VD_3_ inclusion doses on growth performance of growing-finishing pigs.

Items	Inclusion Levels of 25(OH)VD_3_				SEM	*p*-Value		
	0 μg/kg	25 μg/kg	50 μg/kg	75 μg/kg		Diet	Linear	Quadratic
Body weight, kg								
BW d 0	45.16	45.05	45.33	45.32	0.31	0.99	0.99	0.99
BW d 28	71.27	71.49	72.74	72.44	0.73	0.73	0.64	0.61
BW d 56	95.73	96.1	98.79	97.61	1.35	0.61	0.78	0.06
BW d 84	118.92	119.52	122.38	119.6	2.34	0.66	0.69	0.09
ADG, g/d								
d 0–28	933	944	978	969	23.04	0.52	0.48	0.42
d 28–56	873	879	931	899	20.43	0.92	0.72	0.03
d 56–84	828	836	843	785	23.04	0.34	0.57	0.69
d 0–84	878	886	918	884	22.03	0.53	0.76	0.04
ADFI, g/d								
d 0–28	2936	2837	3020	2879	35.34	0.43	0.57	0.34
d 28–56	2699	2618	2719	2656	30.54	0.68	0.43	0.62
d 56–84	3226	3220	3265	3096	35.34	0.43	0.74	0.89
d 0–84	2874	2818	2901	2803	33.23	0.95	0.92	0.84
G: F								
d 0–28	0.315	0.333	0.324	0.337	0.003	0.81	0.69	0.61
d 28–56	0.323	0.336	0.342	0.338	0.004	0.85	0.44	0.54
d 56–84	0.257	0.260	0.258	0.254	0.003	0.85	0.65	0.58
d 0–84	0.305	0.314	0.316	0.315	0.004	0.88	0.83	0.75

Note: Values with different superscript letters within the same row of data indicate significant differences (*p* < 0.05), *n* = 6. 25(OH)VD3, 25-hydroxyvitamin D_3_. ADG, average daily weight gain; ADFI, average daily feed intake; G: F, a ratio ADG to ADFI; SEM, standard error of the mean.

**Table 3:** Effects of 25(OH)VD3 inclusion doses on nutrient digestibility of growing-finishing pigs.

Items, %	Inclusion Levels of 25(OH)VD_3_				SEM	*p*-Value		
	0 μg/kg	25 μg/kg	50 μg/kg	75 μg/kg		Diet	Linear	Quadratic
Dry matter	86.1	85.8	86.9	85.9	0.56	0.55	0.53	0.61
Crude protein	82.9	84.0	83.9	83.0	0.89	0.78	0.57	0.34
Ether extract	53.9	52.8	55.2	53.5	1.81	0.14	0.43	0.23
Calcium	57.9 ^b^	59.8 ^ab^	60.9 ^a^	59.8 ^ab^	0.88	0.01	0.33	0.03
Phosphorus	53.7	54.3	55.0	54.5	1.60	0.76	0.52	0.04

Note: Values with different superscript letters within the same row indicate significant differences (*p* < 0.05), *n* = 6. 25(OH)VD_3_, 25-hydroxyvitamin D_3_. SEM, standard error of the mean.

**Table 4:** Effects of the dietary addition of 0 and 50 µg/kg 25(OH)VD_3_ on the carcass traits and pork quality of growing- finishing pigs.

Items	Inclusion Levels of 25(OH)VD_3_		SEM	*p*-Value
	0 μg/kg	50 μg/kg		
**Carcass traits**				
Hot carcass weight, kg	91.54	91.83	1.6	0.52
Slaughter rate, %	71.42	71.35	0.7	0.73
Carcass slant length, cm	92.00	92.49	0.76	0.31
Backfat thickness, mm	20.90	21.27	0.47	0.17
Eye muscle area, cm^2^	60.61	61.34	1.57	0.45
**Meat quality**				
pH 45 min	6.02	5.91	1.32	0.54
pH 24 h	5.74	5.79	1.03	0.23
Marble Rating	3.17	3.28	0.95	0.65
45 min flesh color				
L*(lightness)	47.21	46.83	4.47	0.42
a*(redness)	18.97	18.10	2.54	0.83
b*(yellowness)	−0.88	−1.01	0.78	0.91
24 h flesh color				
L*(lightness)	51.92	49.94	4.23	0.72
a*(redness)	17.93	17.76	1.44	0.93
b*(yellowness)	3.16	1.92	0.65	0.15
Drip loss, %	2.40	2.59	0.43	0.35
Cooking loss, %	29.65	28.89	2.89	0.46

Note: Values with different superscript letters within the same row indicate significant differences (*p* < 0.05), *n* = 6. 25(OH)VD_3_, 25-hydroxyvitamin D_3_; SEM, standard error of the mean.

**Table 5:** Effects of dietary addition of 0 and 50 µg/kg 25(OH)VD_3_ on amino acid composition in the longissimus dorsi of growing-finishing pigs.

Items, % of the Longissimus Dorsi	Inclusion Levels of 25(OH)VD_3_		SEM	*p*-Value
	0 µg/kg	50 µg/kg		
Lysine	7.62	7.71	0.04	0.12
Methionine	2.57	2.78	0.05	0.14
Threonine	3.91	4.03	0.04	0.78
Tryptophan	0.92	0.99	0.03	0.84
Valine	4.15	4.28	0.06	0.21
Leucine	6.79	6.90	0.04	0.18
Isoleucine	3.96	4.09	0.05	0.31
Phenylalanine	3.33	3.42	0.04	0.41
Histidine	3.94	4.00	0.07	0.43
Arginine	5.32	5.27	0.06	0.34
Tyrosine	2.96	3.05	0.02	0.43
Serine	3.37	3.41	0.04	0.23
Glutamic acid	12.23	12.38	0.04	0.18
Proline	3.11	3.25	0.08	0.43
Glycine	3.56	3.51	0.07	0.23
Alanine	4.73	4.89	0.04	0.35
Cysteine	0.88	0.90	0.06	0.32
Aspartic acid	7.87	8.08	0.12	0.52
Total protein	81.23	82.91	0.53	0.45

Note: Values with different superscript letters within the same row indicate significant differences (*p* < 0.05), *n* = 6. 25(OH)VD_3_, 25-hydroxyvitamin D_3_. SEM, standard error of the mean.

**Table 6:** Effects of the dietary addition of 0 and 50 µg/kg 25(OH)VD_3_ on fatty acid composition of pork longissimus dorsi in growing-finishing pigs.

Items, g/kg	Inclusion Levels of 25(OH)VD_3_		SEM	*p*-Value
	0 µg/kg	50 µg/kg		
C6:0	0.16	0.14	0.02	0.65
C10:0	0.16	0.15	0.02	0.89
C12:0	0.08	0.09	0.01	0.86
C14:0	1.29	1.08	0.11	0.57
C15:0	0.02	0.03	0.01	0.92
C16:0	23.02	19.12	2.05	0.08
C17:0	0.40 ^a^	0.18 ^b^	0.02	0.01
C18:0	11.79	10.39	1.09	0.25
C20:0	0.23	0.19	0.02	0.45
C21:0	0.32	0.38	0.03	0.67
C22:0	0.06	0.08	0.01	0.93
C24:0	0.03	0.04	0	0.92
**Saturated fatty acids**	37.58 ^a^	31.21 ^b^	2.38	0.02
C14:1	0.03	0.02	0.01	0.87
C16:1	3.34 ^a^	2.12 ^b^	0.27	0.04
C18:1n9c	38.47	32.94	3.38	0.35
C20:1	0.73	0.57	0.06	0.24
C24:1	0.08	0.07	0.01	0.85
**Monounsaturated fatty acids**	42.64 ^a^	34.41 ^b^	3.71	0.03
C18:2n6c	7.02 ^b^	9.42 ^a^	0.83	0.04
C18:3n3	0.21	0.40	0.03	0.08
C20:3n6	0.22	0.21	0.02	0.23
C20:4n6	1.48	1.51	0.15	0.54
C20:3n3	0.05	0.06	0.01	0.64
C20:5n3	0.04	0.05	0.01	0.87
C22:1n9	0.03	0.02	0.01	0.94
C22:2	0.05	0.03	0.01	0.96
C22:6n3	0.09	0.09	0.01	0.84
Polyunsaturated fatty acid	9.19 ^b^	11.83 ^a^	1.06	0.01
∑*n*-6PUFA	8.72 ^b^	11.23 ^a^	1	0.02
∑*n*-3PUFA	0.40	0.60	0.05	0.06
*n*-6/*n*-3	22.11 ^a^	17.31 ^b^	1.94	0.02
PUFA/SFA	0.26 ^b^	0.40 ^a^	0.03	0.01

Note: Values with different superscript letters within the same row indicate significant differences (*p* < 0.05), *n* = 6. 25(OH)VD_3_, 25-hydroxyvitamin D_3_. SEM, standard error of the mean.

**Table 7:** Effects of the dietary addition of 0 and 50 µg/kg 25(OH)VD_3_ on the tibia from growing-finishing pigs.

Items	Inclusion Levels of 25(OH)VD_3_		SEM	*p*-Value
	0 µg/kg	50 µg/kg		
Tibia				
Calcium, %	16.76	17.26	0.32	0.09
Phosphorus, %	8.13	8.63	0.45	0.41
Bone mineral content, g	2.93	3.45	0.56	0.87
Bone mineral density, g/cm2	0.33	0.30	0.08	0.94
Break strength, *n*	1510 ^b^	1712 ^a^	92.34	0.01
Destruction deflection, mm	4.21	4.36	0.32	0.67
Stiffness, *n*/mm	582 ^b^	681 ^a^	20.45	0.01

Note: Values with different superscript letters within the same row indicate significant differences (*p* < 0.05), *n* = 6. 25(OH)VD_3_, 25-hydroxyvitamin D_3_.

## Data Availability

The data presented in this study are available on request from the corresponding author.

## Supplementary Materials

The following supporting information can be downloaded at: https://www.mdpi.com/article/10.3390/ani13010086/s1, Table S1: The primer sequences of antioxidase and calcium ion channel protein genes.
