# Supplementary material for: Effects of 25(OH)VD3 on Growth Performance, Pork Quality and Calcium Deposit in Growing-Finishing Pigs
Source: Animals (Basel). 2022 Dec 26;13(1):86. doi: 10.3390/ani13010086 (PMC9817873; doi:10.3390/ani13010086)
Supplement: Supplementary file 1 [file animals-13-00086-s001.zip › animals-2060611-supplementary.pdf]

Supplementary Table S1. The primer sequences of antioxidase and calcium ion channel protein genes

Genes	Primer sequences Primersequences(5'to3')	Size(bp)	Accession No.
Cu/Zn-SOD	F:AACCAGATGACTTGGGCAGA<br>R:AGACCATGGCATGAGGGAAT	120	NM_001190422.1
CAT	F:AGATGGACACAGGCACATGA<br>R:TTGATGCCCTGGTCAGTCTT	111	NM_214301.2
GPx1	F:GGTTCGAGCCCAACTTCATG<br>R:CATTGCGACACACTGGAGAC	165	NM_214201.1
VDR	F: AGGCTTCTTCAGACGGAGCATGAA<br>R: ACTCCTTCATCATGCCGATGTCCA	200	XM_021091108.1
TRPVD6	F: CACTGGGTGTCCCAAAGTCC<br>R: ACTGGCCAGACACAGAGACT	292	XM_021078898.1
CaBP-D28k	F: TGAGCTTTTGCTCACTCCCC<br>R: ACTTCCGTCAGCGTCGAAAT	162	NM_004929.3
CaBP-D9k	F: GCTTCAGACGGAATTCCCCA<br>R: TCCATCACCGTTCTTATCCAGT	85	HQ_331533.1
GAPDH	F: TCGGAGTGAACGGATTTGGC<br>R: TGACAAGCTTCCCGTTCTCC	189	NM_001206359.1
